# Thalamo-peduncular germinoma in a young adult: a case report

**DOI:** 10.11604/pamj.2026.53.109.50181

**Published:** 2026-03-03

**Authors:** Mohamed Saadoune, Khaoula Ait Benkacem, Samir Barkiche, Mouna Khouchani

**Affiliations:** 1 Service of Radiation Oncology/Morpho-science Laboratory, University Cadi Ayyad of Marrakech, Marrakech, Morocco,; 2 University Hospital Mohammed VI of Marrakech, Faculty of Medicine and Pharmacy of Marrakech, Marrakech, Morocco

**Keywords:** Intracranial germ cell tumor, magnetic resonance imaging, stereotactic biopsy, thalamo-peduncular, case report

## Abstract

Germinomas arising in deep brain structures such as the thalamus and cerebral peduncles are rare and frequently mimic high-grade gliomas, creating diagnostic uncertainty. We report the case of a 23-year-old man presenting with progressive headaches, visual disturbances, and signs of intracranial hypertension with secondary hemiparesis and speech impairment. Brain magnetic resonance imaging (MRI) showed a thalamo-peduncular mass suggestive of infiltrative glioma. Stereotactic biopsy with immunohistochemistry demonstrated strong CD117, PLAP, and OCT4 expression, confirming a pure germinoma. Staging revealed pulmonary micronodules and para-aortic lymphadenopathy, while serum tumor markers were normal. The patient was treated with platinum-based chemotherapy followed by craniospinal radiotherapy, with marked clinical improvement and radiological stability. This case illustrates the diagnostic challenge of deep midline germinomas and emphasizes the essential role of histological confirmation to ensure appropriate, potentially curative treatment.

## Introduction

Intracranial germ cell tumors (IGCTs) are rare neoplasms arising from primordial germ cells that aberrantly migrate to the central nervous system, accounting for less than 5% of primary brain tumors in Western populations and up to 10% in East Asian countries [1]. They predominantly affect children and adolescents, with a marked male predominance, and typically involve midline structures such as the pineal gland or suprasellar region [2]. Germinomas represent the most frequent histological subtype and generally exhibit a favorable prognosis when appropriately treated. However, their clinical presentation is highly variable and often influenced by the lesion´s anatomical location, leading to heterogeneous and sometimes misleading manifestations.

Deep-seated germinomas arising in the thalamus or thalamo-peduncular region are exceptionally uncommon, representing a small minority of intracranial germinomas [3]. Their atypical location frequently results in nonspecific symptoms such as progressive hemiparesis, cognitive disturbances, or speech impairment, which may initially suggest alternative diagnoses. Radiologically, these tumors pose a significant challenge, as their MRI characteristics frequently overlap with those of infiltrative high-grade gliomas, demyelinating diseases, or inflammatory processes [3]. The absence of elevated serum tumor markers in many cases further contributes to diagnostic uncertainty, delaying appropriate management. In low- and middle-income settings, including many African countries, limited access to advanced neuroimaging and specialized neuropathology services may exacerbate diagnostic delays and misclassification.

Because germinomas are highly radiosensitive and chemosensitive, timely and accurate diagnosis is essential to ensure optimal therapeutic outcomes and avoid unnecessarily aggressive surgical interventions [4]. Histopathological confirmation through stereotactic biopsy, combined with immunohistochemical markers such as CD117, PLAP, and OCT4, remains the cornerstone for definitive diagnosis and treatment planning [4]. Reports of thalamic or thalamo-peduncular germinomas from Africa remain extremely scarce, making awareness of this entity crucial for clinicians practicing in the region. We report a rare case of thalamo-peduncular germinoma in a young adult initially misdiagnosed as a high-grade glioma, highlighting the importance of tissue diagnosis and immunohistochemical evaluation in distinguishing these tumors from other deep-seated intracranial lesions.

## Patient and observation

**Patient information:** a 23-year-old man with no significant medical history presented in May 2022 with gradually worsening headaches, visual disturbances, and symptoms suggestive of intracranial hypertension. Over the following weeks, his condition progressively deteriorated, leading to right upper limb functional impairment, hemiparesis, dysarthria, and left-sided ptosis. There was no history of infection, trauma, or exposure to toxic substances, and no relevant familial neurological or oncological background was reported.

**Timeline of current episode:** the patient's symptoms began insidiously with headaches and visual disturbances, progressively worsening over several weeks. Neurological deficits subsequently developed, including motor weakness and speech difficulties, prompting further investigation. Diagnostic imaging was performed soon after presentation, followed by stereotactic biopsy and initiation of systemic treatment. Chemotherapy was administered over four cycles, after which radiotherapy was delivered, and follow-up imaging confirmed disease control.

**Clinical findings:** on clinical examination, the patient exhibited signs of intracranial hypertension associated with progressive neurological deficits. Findings included right-sided hemiparesis affecting the upper limb predominantly, dysarthria, and left-sided ptosis. Coordination and motor function were significantly impaired, consistent with involvement of deep brain structures. No systemic abnormalities were noted.

**Figure 1:**
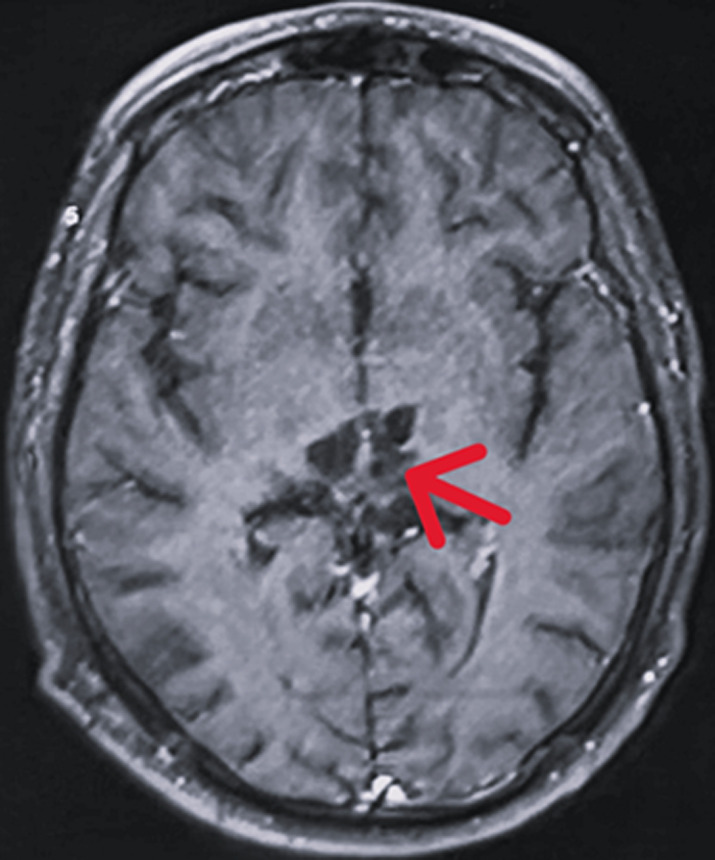
contrast-enhanced brain magnetic resonance image showing a thalamo-peduncular lesion: axial T1-weighted post-contrast magnetic resonance image demonstrating a thalamo-peduncular mass with heterogeneous patchy enhancement and multiple small cystic components (red arrow)

**Figure 2:**
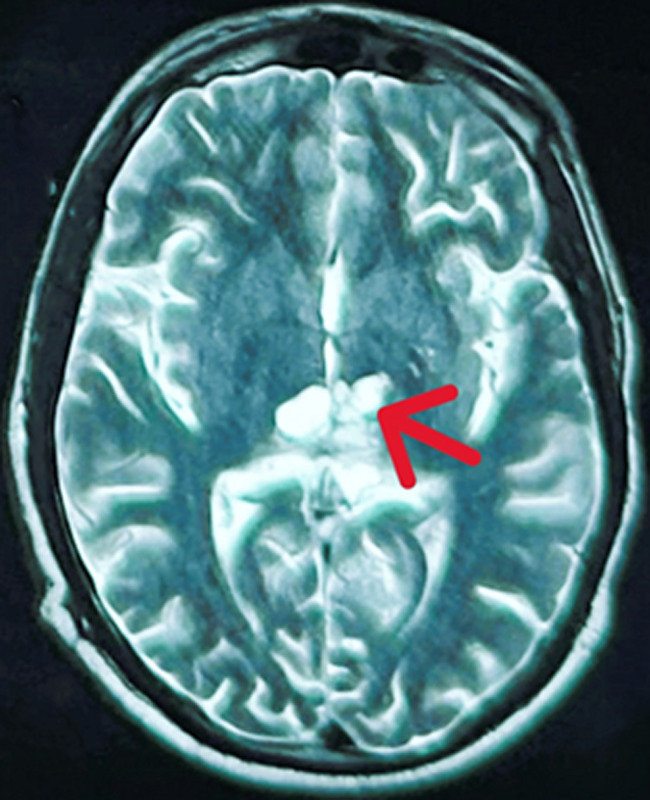
axial T2-weighted brain magnetic resonance image showing a thalamo-peduncular mass: axial T2-weighted magnetic resonance image demonstrating a hyperintense lesion in the thalamo-peduncular region with surrounding edema and compression of the third ventricle (red arrow)

**Figure 3:**
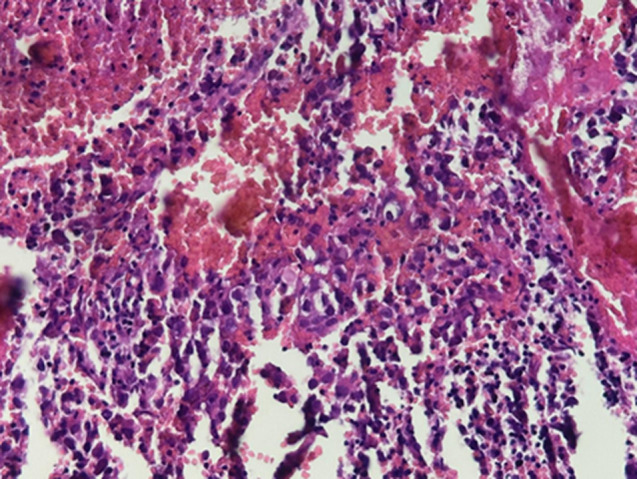
histopathological appearance of the tumor on hematoxylin-eosin staining: hematoxylin-eosin stained section showing a population of tumor cells with round hyperchromatic nuclei arranged in diffuse sheets (original magnification x20)

**Figure 4:**
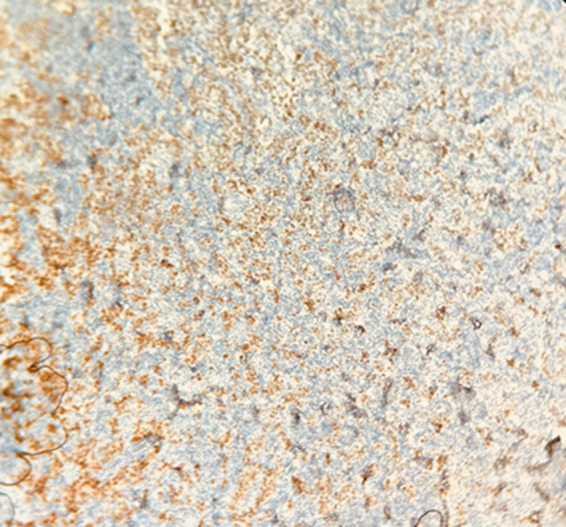
immunohistochemical nuclear staining of tumor cells supporting germinoma: immunohistochemical staining showing strong nuclear positivity of tumor cells for OCT4, consistent with the diagnosis of germinoma

**Figure 5:**
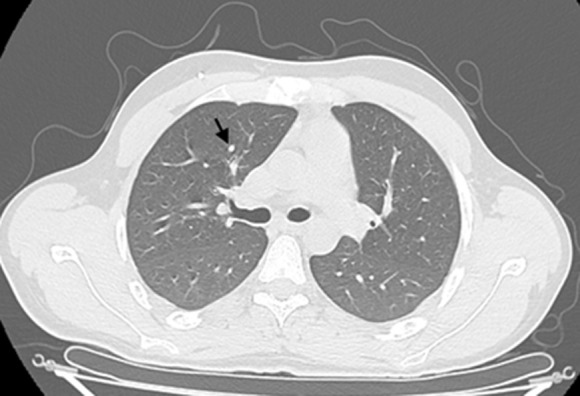
chest computed tomography image showing a non-specific pulmonary micronodule: parenchymal window chest computed tomography image demonstrating a small non-specific pulmonary micronodule (arrow) without associated consolidation or mass effect

**Diagnostic assessment:** magnetic resonance imaging of the brain demonstrated an infiltrative thalamo-peduncular lesion with hyperintensity on T2/FLAIR sequences and faint enhancement on post-contrast T1, raising concern for an infiltrative high-grade glioma and creating diagnostic uncertainty (Figure 1, Figure 2). A stereotactic biopsy was subsequently performed. Initial histological evaluation suggested a high-grade glioma; however, immunohistochemical staining revealed strong positivity for CD117, PLAP, and OCT4, findings consistent with a pure germinoma (Figure 3, Figure 4). Staging with thoraco-abdominopelvic CT identified nonspecific pulmonary micronodules (Figure 5), while serum tumor markers remained within normal limits (Table 1). As shown above, the patient's LDH level is elevated compared to the reference range, while both β-HCG and α-FP are within normal limits. These values are important for the diagnostic evaluation and ongoing management of intracranial germ cell tumors.

**Table 1:** biological test results

Parameter	Patient result	Reference values
LDH (lactate dehydrogenase)	259 (U/L)	135–225 (U/L)
β-HCG (beta-human chorionic gonadotropin)	< 2 (U/L)	< 5 (U/L)
α-FP (alpha-fetoprotein)	1.21 (ng/mL)	< 10 (ng/mL)

**Diagnosis:** based on radiological features and immunohistochemical analysis, the final diagnosis of a pure thalamo-peduncular germinoma was established.

**Therapeutic interventions:** the patient received four cycles of chemotherapy. The first and third cycles included etoposide and carboplatin, while the second and fourth cycles combined etoposide and ifosfamide. Following systemic treatment, he underwent craniospinal irradiation at a total dose of 24 Gy. No major treatment-related complications were reported.

**Figure 6:**
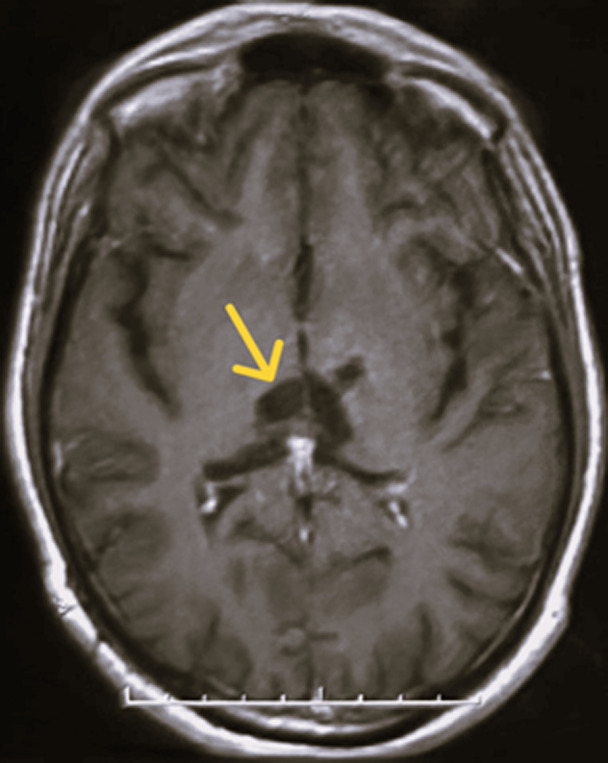
post-treatment contrast-enhanced brain magnetic resonance image showing regression of the thalamo-peduncular lesion: axial T1-weighted post-contrast magnetic resonance image obtained after treatment demonstrating cystic regression of the lesion with absence of residual contrast enhancement (yellow arrow)

**Figure 7:**
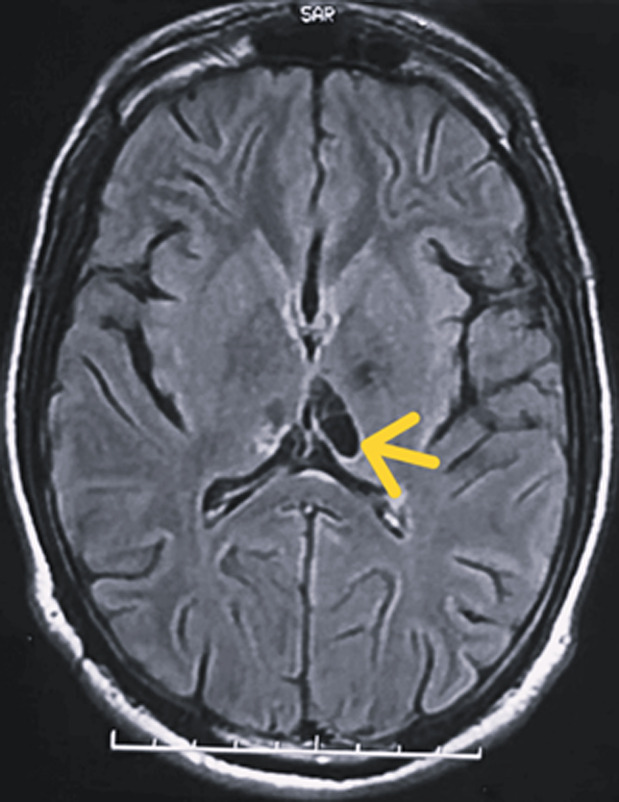
axial brain magnetic resonance image showing post-treatment gliotic changes: axial fluid-attenuated inversion recovery magnetic resonance sequence obtained after radiotherapy demonstrating gliotic signal changes and collapse of the third ventricle (yellow arrow)

**Follow-up and outcome of interventions:** clinical reassessment after completion of therapy revealed marked improvement in coordination, speech, and motor function. Follow-up MRI demonstrated a stable multicystic lesion surrounded by marginal gliosis, with collapsed lateral ventricles suggestive of hyperdrainage, without evidence of progression (Figure 6, Figure 7). The patient remained clinically stable on subsequent evaluations.

**Patient perspective:** the patient reported significant improvement in daily functioning and expressed relief after receiving a definitive diagnosis and effective treatment. He highlighted the major impact of motor and speech recovery on his quality of life.

**Informed consent:** written informed consent was obtained from the patient for publication of this case report and accompanying images.

## Discussion

Germinomas of the thalamo-peduncular and basal ganglia regions are an uncommon and diagnostically challenging subset of intracranial germ cell tumors, representing less than 10% of all central nervous system germinomas [5]. Their deep location, combined with non-specific clinical manifestations, often leads to delayed or incorrect diagnosis [6]. Unlike pineal and suprasellar germinomas, which are more readily recognized due to their typical midline presentation, these ectopic lesions may mimic high-grade gliomas, demyelinating conditions, or inflammatory disorders [7]. The insidious onset of symptoms such as hemiparesis, cognitive decline, movement disorders, and raised intracranial pressure complicates the clinical picture, as seen in our patient. Awareness of these atypical presentations is critical to avoid mismanagement and ensure timely biopsy [8].

Neuroimaging remains the first step in evaluation, but has inherent limitations. Magnetic resonance imaging features of germinomas in these locations often include T2/FLAIR hyperintensity, variable gadolinium enhancement, and spectroscopy abnormalities such as elevated choline and reduced NAA peaks [8]. However, these characteristics significantly overlap with high-grade gliomas and other infiltrative tumors, explaining the initial misdiagnosis in many cases [9]. Several authors suggest that unilateral basal ganglia or thalamic lesions in adolescents or young adults, especially when associated with progressive hemiparesis and normal tumor markers, should raise suspicion of germinoma [10]. Ultimately, histopathological examination with immunohistochemistry remains the diagnostic gold standard, with typical markers including CD117, PLAP, and OCT4.

Therapeutic management of intracranial germinomas has evolved to optimize survival while minimizing long-term toxicity. Multimodal treatment using platinum-based chemotherapy combined with radiotherapy has demonstrated excellent outcomes across pediatric and young adult populations [10]. In our patient, this combined approach led to significant clinical and radiological improvement, mirroring previously published outcomes. Reports of thalamic or thalamo-peduncular germinomas from Africa remain extremely scarce, which may contribute to diagnostic delays in resource-limited settings. Long-term sequelae of therapy remain an important concern, particularly regarding neurocognitive function and neurological deficits related to both tumor involvement and treatment effects. Consequently, close multidisciplinary follow-up is essential to monitor functional recovery, detect recurrence, and address late toxicities. In summary, thalamo-peduncular germinomas, although rare, must be considered in the differential diagnosis of deep-seated brain tumors in young adults. Early biopsy with immunohistochemistry is critical to distinguish them from more aggressive neoplasms, and multimodal treatment continues to offer favorable outcomes despite the challenges posed by their unusual location.

## Conclusion

This case of thalamo-peduncular germinoma in a young adult, initially suspected to be a high-grade glioma on imaging, illustrates the major diagnostic pitfall posed by deep midline tumors. The key lesson is that early stereotactic biopsy with immunohistochemical analysis is crucial to establish the correct diagnosis and guide appropriate chemoradiotherapy, thereby avoiding misclassification and inappropriate management.
